# How far has the digitization of medical teaching progressed in times of COVID-19? A multinational survey among medical students and lecturers in German-speaking central Europe

**DOI:** 10.1186/s12909-022-03470-z

**Published:** 2022-05-20

**Authors:** Stefan Ferdinand Hertling, David Alexander Back, Niklas Eckhart, Mario Kaiser, Isabel Graul

**Affiliations:** 1 Department of Obstetrics and Gynaecology, University Hospital Jena, Jena, Germany; 2 Clinic for Traumatology and Orthopedics, Bundeswehr Hospital Berlin, Berlin, Germany; 3 Dieter Scheffner Center for Medical Education and Educational Research, Charité – Universitätsmedizin Berlin, Berlin, Germany; 4 Institute for Diagnostic and Interventionel Radiology, University Hospital Jena, Jena, Germany; 5 modul integration optics, Jenoptik Light & Optics Devision, Jena, Germany; 6 Department of Orthopedic and Trauma Surgery, Martin-Luther University Halle-Wittenberg, Ernst-Grube-Straße 40, 06120 Halle (Saale), Germany; 7 Department of Trauma-, Hand- and Reconstructive Surgery, University of Jena, Jena, Germany

**Keywords:** Digitization, Medical education, Survey, Undergraduate medical education, Online learning, Virtual learning

## Abstract

**Background:**

To ensure successful medical education despite the COVID-19 pandemic, the demand for online instruction has substantially increased. Fast and efficient teaching in a digital format poses a great challenge for medical students and lecturers as well as the universities.

**Objective:**

The aim of this study is to capture the readiness of medical students and faculty members to participate in rapidly- evolving online education.

**Methods:**

This cross-sectional study is based on two questionnaires distributed among medical students and associate deans for education in Germany, Austria and Switzerland. Questions included decision- making questions, categorical questions, and open-ended questions, all addressing the frequency and format of the digital education offered, the perceived quality of digital education, and medical student satisfaction with digital education. Questions about missing content and areas for improvement from the perspectives of medical students were included. The associate deans were asked for their opinions about the impact of the pandemic on teaching, the organizational setup and implementation of digital education by universities, and plans for future initiatives.

**Results:**

Three thousand and thirty medical students (m = 752 and f = 2245) from 53 universities participated in the study. The study showed that 92% of students were affected by the pandemic, and 19% of the students viewed the changes as entirely negative. 97% of the medical students were able to participate in digital courses, but only 4% were able to learn exclusively online. For 77% of the medical students, digital offerings accounted for over 80% of the education offered. In terms of content, medical students complained about a lack of practical teaching, such as contact with patients, lecturers, fellow medical students, and a poor perceived quality of teaching due to dubbing, frequent changeover of seminars, problem-oriented learning groups and in-person teaching, a lack of interaction possibilities and a lack of technical equipment, such as lecturers’ knowledge and server capacities, at the universities. Overall, almost half of the medical students (42%) rated the implementation of digital teaching at their universities as good or very good. Forty-one of the 53 associate deans responded to the questionnaire, and 35 felt medical education was influenced by the pandemic. The associate deans (80%; 33/41) felt that the digitalization of medical education was negatively influenced by the pandemic. Only 44% (18/41) felt that their universities were well or very well positioned for digital teaching. All the associate deans believe that digital teaching in medicine will continue after the pandemic.

**Conclusions:**

In the German-speaking world, the rapid conversion of medical teaching to a digital format has been well implemented in many cases. The perceived quality of the implementation of digital education still lacks practical relevance and the use of new digital media, such as learning games, VR, and online question time. The digital format of medical education will likely continue beyond the COVID-19 pandemic.

## Introduction

The COVID-19 pandemic has drastically changed countless aspects of daily life around the world. For instance, the COVID-19 pandemic has led to an unprecedented restructuring of medical education worldwide. Digital platforms had to be made available by faculty members in a very short time to ensure learning. Lectures, seminars, and clinical internships could no longer be conducted in familiar face-to-face settings. Medical educators worldwide have discussed the possibility of entirely eliminating all student activities involving direct patient contact [1]. If medical educators wish to continue to deliver quality medical education during the COVID-19 pandemic, they must overcome the challenges of the pandemic [2]. Suggested solutions have included digital technologies to support innovative teaching on e-learning platforms, virtual training, and videoconferencing [2–5]. Therefore, the medical education curriculum needs to be developed to provide medical students with opportunities for continuous learning and avoid delays due to the pandemic [6]. Although the pandemic appears to be an unusual catalyst for the promotion of eLearning, it is still unclear whether medical students believe they are being offered a sufficient online format that is comparable to face-to-face teaching in terms of quantity and knowledge transfer in order to successfully complete their medical education. Digital teaching potentially offers many advantages in terms of flexibility and convenience in meeting learning needs, reducing overall costs, communicating efficiently with medical students and incorporating their feedback [7, 8]. In the published literature, the findings of assessments of digital medical education appear to be contradictory. For example, Singh et al. showed that in India, only 20.4% felt e-learning can replace conventional teaching [9]. For some other authors, satisfaction with digital learning was shown to be equal to or better than satisfaction with face-to-face instructional offerings [10–12].

Information on how medical education has changed as a result of the pandemic in German-speaking central Europe is lacking. The number of various teaching formats that have been digitized and the form into which they have been digitized are unclear. The value assessment of associate deans and students involved in the process of pivoting from conventional face-to-face teaching/learning to a digital format has not yet been investigated.

In this study, an overview of the use of digital media teaching by medical faculties in German-speaking central Europe should be provided. For this purpose, the quantity and quality of virtual education perceived by medical students and lecturers was recorded in an online survey. The teachers and medical students were surveyed to determine their readiness to participate in and attitudes toward online education to potentially accelerate the adoption and implementation of this teaching method. This study serves to illustrate the evolution of medical teaching throughout the pandemic in German speaking central Europe. In a further step, the study will be useful for establishing digitization in medical teaching within a defined framework.

## Methods

Two surveys were conducted among medical students and the faculty on the perceived quality and quantity of medical teaching during the COVID-19 pandemic. Participation in the study was voluntary. Before the study, all the participants provided informed consent. The responsible local ethics committee was informed and had no objections to the study (Reg.-Nr.:2019-1456-Bef).

### Questionnaire design

The study questionnaires were designed following published guidelines for questionnaire-based research on a web-based design [13–15]. The selection of questions for the questionnaire was based both on similar studies and on the quality criteria for online questionnaires [16]. The surveys were created in SurveyMonkey™ (SurveyMonkey, San Mateo, CA).

### Survey performance

The medical student survey was distributed among all medical student councils at the 53 medical schools in Germany, Austria and Switzerland. The faculty questionnaire was distributed among all associate deans of teaching. Associate deans with teaching responsibilities were selected to comprehensively assess the quantity of digital teaching and thus to present their perceived quality and quantity of the university’s digital medical education without distortion. The duration of survey data collection was from November 2020 to February 2021. Medical students at all stages of their studies were included. In these countries, the study program is divided into 3 sections: 2 years of preclinical basic studies, 3 years of clinical studies and a practicum year in the clinic. With a population of 117,000 medical students, a confidence interval of 95% and an error margin of 2.5, the target case number was 2,925. Thus, the online survey can be considered representative of the medical student population in Germany, Austria and Switzerland.

The questionnaires were distributed electronically via email lists from the medical student councils to all enrolled medical students and all associate deans.

In an information letter, participants were informed that their data would be strictly treated as confidential and anonymized. Access to the study was granted with a survey link.

### Medical student questionnaire design

A 27-item, self-administered online questionnaire survey was developed with a comprehensive list of questions based on published research on digitalization among medical students [17–19]. Members of the Working Group Digitalization of the DGOU (German Society for Orthopaedics and Trauma) were invited to review the validation process and provide feedback on the question format, comprehensiveness, clarity, and flow [20]. The questionnaire was refined according to their input. The questionnaire consisted of binomial questions, questions on a categorical 5- point Likert scale and open-ended questions and was entitled “digitalization among medical students”. The main sections were:

1. Respondent demographics: Epidemiological data on gender (male, female, gender neutral), semester level (preclinical medicine, clinical medicine, practicum year and others, free semester or vacation semester), age and university location (open-ended).
2. Influence of the pandemic on current lessons: Is there an impact (Yes/No)? How is medical education impacted (positively, negatively, both, not at all)?
3. The university’s approach to teaching in the pandemic: Overall assessment of how the university is addressing current situation (rated on a 5 -point Likert scale: not at all, not so good, average, good, fully addressed), whether face-to-face teaching is currently available (Yes/No) and what kind of teaching is available (multiple choice: lecture, practicums, seminars, bedside teaching, exams, tutorials), whether digital teaching is currently available (Yes/No) and the format of this education (multiple choice: lecture, seminar, exams, tutorials, electives, teaching opportunities for exam preparation), and what examination modalities are available (digital, attendance, both)?
4. Assessment of digital teaching: Assessment of the relevance of exam preparation (rated on a 5- point Likert scale: not at all, a little, moderately, good, completely), and percentage of education is digital teaching (10 choices ranging from 10 to 100%)?
5. Implementation of digital teaching: whether additional technical equipment is available (Yes/No), what types of digital platforms are available (“ZOOM”, “WebEx”, “Adobe”, Teams”, other, none), assessment of the accessibility of the instructor (better, same, worse), whether learning management systems are used (Yes/No), and which learning management systems are in use (Moodle, Blackboard, university-owned), and whether practical teaching content is included (Yes/No)?
6. Assessment of the implementation of digital teaching: Assessment of user-friendliness of the media offered (not at all, a little, moderate, good, complete), satisfaction with the organization (not at all, a little, moderate, good, complete), desire for more practice-oriented digital teaching (Yes/No), what content is available (open-ended), assessment of the university’s setup for the application of digital teaching (Yes/No), and suggestions for improvement in digital teaching (open-ended).

### Study questionnaire contents for medical faculties

Referring to the medical student survey, a faculty survey was designed with 7 items. The survey consisted of two ordinal questions, one 5- point Likert-scale question, and 4 open-ended questions.

1. Influence of the pandemic on medical teaching at one’s own university.

Does the COVID-19 pandemic affect medical education for medical students (Yes/No), and how is it affected (open-ended)? How are courses currently conducted (face-to-face, digital, both). How well is your university positioned for the transition to digital teaching (5 -point Likert scale).

2. Assessment of the overall development of medical teaching.

Is the COVID pandemic influencing the digitization of medical education (open-ended)? Due to the COVID-19 pandemic, will the learning content relevant to the exam will be more difficult/less frequently taught (open-ended)? Will digital teaching tools remain an important part of medical education even after the COVID-19 pandemic (open-ended)?

Both surveys were of short duration, a maximum of ten minutes to minimize the drop-out rate as much as possible and to maximize the motivation to answer the questions [21, 22]. We conducted an abductive analysis of the interview transcripts and the results of the open-ended questions of the questionnaire from faculty and students to identify the predominant themes. The theme consisted of several codes, which were later defined as subthemes. Saturation of the codes was achieved with the three interviews to ensure that most aspects related to our research question were covered. All coding was conducted and reviewed by IG and SH using MaxQDA qualitative data analysis software (MaxQDA 18.3.2; VERBI GmbH).

### Data analysis

Only fully completed questionnaires were included in the analysis. The results were compiled using SurveyMonkey™ and the Statistical Package for the Social Sciences (SPSS) (version 17.0, SPSS Inc. Chicago, IL, USA). The numbers of students or lecturers were given as percentages and absolute numbers in brackets.

## Results

### Medical student survey

Of the 117,000 students enrolled at medical schools in Germany, Austria and Switzerland (in the winter semester of 2019/2020), 3030 responded to the survey. Differences between countries have not been analyzed; although there are similar education systems, there are also many differences. This distinction was not the aim of the work Respondent demographics.

Overall, 25% (752/3030) of the participants were male, 74% were female (2247/3030) and 1% were of gender neutral (31/3030). Responses were received from medical students in the early semesters (first two years of preclinical medical studies; 7%, n = 200/3030), from the later semesters (three years of clinical medical studies; 68%, n = 2052/3030), from the practicum year (22%, n = 671/3030), and the remaining 3% (n = 107/3030) of respondents were not in a defined semester (pregnancy, vacation semester, PhD thesis). Responses from all 53 universities were included, with a minimum of 0.3% (10/3030) and a maximum of 8.8% (265/3030) of respondents from the same university. In all semesters, more female medical students answered the questionnaire. The highest participation rate of 68% (2052/3030) was recorded in the clinical study phase. German medical students made up the majority of the participants (68%; 2067/3030), but Germany also had the highest number of medical universities (79%; 42/53). The demographic data are shown in Table 1. Differences between countries were not analyzed with similar education systems.

**Table 1:** Demographic data of the medical students

Students	Female	Male	Gender neutral
3030	2247	752	31
Preclinical semester (200)	105	93	2
Clinical semester (2052)	1576	470	6
Practicum year (671)	508	148	23
others (107)	58	41	8
Country	Germany	Switzerland	Austria
	2067	567	396
Universities (53)	42	7	4

#### Influence of the pandemic

A majority of medical students found their medical educations affected by the pandemic (92%; 2789/3030). When asked how the pandemic affected their education, whether positively, negatively, or both, 73% (2217/3030) responded “both”, with 19% (578/3030) reporting that the pandemic had a negative impact.

#### The university’s approach to teaching during the pandemic

The majority of medical students (59%; 1794/3030) felt that the university’s handling of the pandemic situation was rather average. 81% (2468/3030) of respondents indicated that face-to-face events were still taking place. These primarily included bedside teaching, reported by 77% of students (1898/2468) and internships, reported by 58% of students (1427/2468). Examinations still occurred in person, according to 36% of respondents (881/2468), 30% of respondents (751/2468) reported attending seminars in person and 29% (724/2468) reported attending lectures. 17% of students (416/2468) reported that tutorials were held in person. 97% (2928/3030) of universities offered digital instruction. Lectures (79%; 2304/2928) and electives (75%; 2202/2304) were offered digitally in three-quarters of the universities, while seminars (69%; 2007/2928) and courses for exam preparation (67%; 1951/2928) were offered in two-thirds of the universities, exams were offered in 63% of the universities (1845/2928), and tutorials were offered in 59% of the universities (1737/2928). In terms of exam modalities, 52% (1576/3030) indicated digital and in person; only 4% (128/3030) were fully digital.

The main criticism of medical students was the lack of clinical practice, such as hands-on suture techniques, sonography, and bandage techniques.

#### Medicine is a hands-on profession and requires the acquisition of medical skills through patient examination

In particular, contact with patients was lacking for many medical students. Medical students desired a more in-depth study of examination methods, surgical techniques and diagnostic criteria using case studies and discussions. These problem-oriented approaches in small groups help to provide a link between theory and practice.

“*I lack contact with patients with real responses and reactions, learining a theoretical physical examination of a patient is difficult.*” (medical student, female, clinical semester)

Many practicum courses and seminars were offered as online lectures.

Many of the online lectures were only available as PowerPoints or PDFs without dubbing or with asynchronous dubbing. The prerecorded digital content provided no opportunity for questions or discussion. As a result, medical students criticized the impersonal approach and felt left alone with the problem of acquiring exam-relevant content.

“*The digital formats are almost exclusively teaching without interaction possibilities*.” (medical student, female, clinical semester)

#### Assessment of digital teaching

The medical students were asked for their opinions on the relevance of the current instruction to their examinations. More than half (60%; 1820/3030) perceived the relationship as moderately relevant on 5 point Likert scale (not at all: 1%, 23/3030; a little: 4%, 135/3030; moderate: 60%, 1820/3030, good: 32%, 978/3030; completely: 1%, 35/3030). Among the 3030 students surveyed, the most common response (n = 1212;40%) was that their instruction was 90% in digital form. Under 10% (n = 203; 7%) of students (203/2997) have under 70% digital instruction.

An overwhelming proportion of medical students criticized the lack of planning and structure. The digital formats were often online with a time delay, and the formats varied depending on the lecturer.

“*If I at least knew when a lecture was online or a class was taking place digitally, I could schedule that, but mostly face-to-face classes are scheduled, which are cancelled*.” (Student, female, clinical semester)

#### Implementation of digital teaching

According to 89% of students (2699/3030), electronic devices used to participate in digital offerings were available. In 83% (44/53) of the universities, the necessary electronic equipment was provided. For the implementation of digital teaching, a digital web-based platform was used: Zoom in 70% of all cases (2112/3030), Teams in 13% (396/3030), WebEx in 10% (314/3030), Adobe Connect 2% (57/3030) and others in 3% (100/3030). The accessibility of the lecturers during the COVID-19 pandemic was assessed by three-quarters of students as unchanged (76%; 2315/3030) and worse by 14% of students (439/3030).

According to a majority of medical students, 85% of their universities (2578/3030) offered learning management systems. These were university internal systems in 90% of cases (2739/3030), Moodle in 7% of cases (221/3030), and Blackboard in 1% of cases (45/3030). Medical students also indicated that hands-on content, such as case studies, surgical techniques, and examination techniques, was taught digitally 91% of the time (2763/3030).

#### Assessment of the implementation of digital teaching

Medical students perceived ease of use to be moderate to good on a 5- point Likert scale. The medical students rated the user-friendliness of the media offered (not at all: 0.3%, 10/3030; little: 3%, 81/3030; moderate: 53%, 1611/3030; good: 41%, 1246/3030; complete: 1%, 37/3030), and they rated satisfaction with the organization of digital teaching (not at all: 1%, 33/3030; little: 6%, 180/3030; moderate: 64%, 1926/3030; good: 27%, 831/3030; complete: 0.5%, 15/3030) as average to good on a 5 point Likert scale. Medical students desired more hands-on teaching, according to 89% of respondents (2686/3030). The open question about which content the medical students would like to see more of is shown in Table 2. An overwhelming majority of medical students (89%; 2706/3030) believe their university is well positioned to apply digital teaching concepts.

**Table 2:** Overview of the points complained about by the students about digital teaching

Main topic	Problem	Details
Digital infrastructure	Server capacities	
	Central learning platform	
	Organization	Planning reliablility
		Structuring
		Unification of formats
Feedback mechanismen	Self-control	Sucess checks (MC questions)
		Educational games
	External control	Small groups via platforms
		Online consultation with the lecturers
Content	Practice	Diagnostic algorithm
		Operation technique
		Patient examination
		Plaster course
		Seam course
		Sonographic course
	Linkage with practice	Interactive event
		Virtual reality
		Simulation
		Videos at the bedside
Quality of digital teaching	Availability on platforms	
	Lecture synchronization	
	Interactive forms of teaching	Internships
		Seminars
		Small groups teaching case studies

To achieve a more hands-on approach, medical students would like to see opportunities for interactive participation, such as online office hours, faculty question-and-answer sessions, and interactive opportunities through simulations and the use of virtual reality. The possibility of receiving feedback seemed to play a major role for many medical students, for example, in the form of responses by lecturers or fellow medical students in Zoom meetings or as self-monitoring with digital knowledge tests.

“*I would like to have the opportunity to do an internship or seminar interactively, gladly in a small group with the possibility to get feedback*.” (Student, male, clinical semester)

### Associate dean survey

#### Influence of the pandemic on medical teaching at the university

Forty-one associate deans of teaching responded to the survey, and 85% (35/41) affirmed that the COVID-19 pandemic affected medical students’ education. In response to the open-ended question about the effect of the pandemic on medical education, several noted the small number of class sessions, the lack of knowledge, as well as fewer opportunities for hands-on learning with active contact with patients, faculty, and fellow medical students as challenging.

“*Students have less time in the affected acute care disciplines with patient contact and fewer opportunities for preparation in training and participation in rounds, for example, due to the limited number of people in the rooms*.“ (Sample response from an associate dean)

When asked about how they currently deliver university-based courses, 59% of associate deans (24/41) responded that classes were both in digital and face-to-face formats. Only 39% (16/41) stated that they offer only digital teaching, and an associate dean reported that this university continues to teach face-to-face with adherence to sanitation standards. The associate deans were asked how they would rate their ta.??? A relatively small percentage of the deans (15%; 6/41) complained of poor preparation for virtual teaching, 41% (17/41) rated it as average, 29% (12/41) as good, and 15% (6/41) as very good.

“*In a sense, more pressure is being created to drive digitization forward. However, the means and the corresponding expertise must first be available; something like this doesn’t happen overnight*.” (example response from an associate dean)

#### Assessment of the overall development of medical teaching

When asked whether the associate deans see the COVID-19 pandemic as requiring digitization in medical teaching, 78% (32/41) answered with a resounding yes. In the open-ended question, criticism was often raised that the short time required for the conversion to digital teaching and ad hoc implementation were often associated with a reduction in the quantity of teaching. Some deans also complained about the poor technical equipment at their universities and the lack of skills to fully use digital media.

“*There should be an extra subject for digital medicine*.” (example response from an associate dean).

71% (30/41) of the associate deans surveyed felt that it was more difficult to convey knowledge that was relevant to examinations when teaching virtually. In the case of knowledge relevant to exams, it is primarily the practicum content that medical students lack and that cannot be completely taught virtually. The opinion of associate deans on the future development of teaching is homogeneous; digital teaching will continue to be a more important part of medical education after the pandemic.

## Discussion

This multinational survey of medical students enrolled in medical schools in Germany, Austria and Switzerland in the 2020/2021 winter semester was designed to provide an overview of virtual instruction and to evaluate the views of those affected. The digitalization of medical teaching has been increased by the pandemic. Digital teaching formats are now more common and are often available to 97% of the medical students surveyed. Nevertheless, the medical students felt that the university’s response to the pandemic was only moderate, and satisfaction with digital teaching was also evaluated as moderate. The foundations for digital teaching have been laid, but there are still many opportunities to build on them.

92% of medical students and over two-thirds of associate deans felt medical teaching was impacted by the COVID-19 pandemic. Interestingly, only 19% of the surveyed medical students perceived the impact as completely negative. The positive aspects of the pandemic on medical teaching were not explicitly recorded. The open-ended questions suggest a large proportion of positive effects of the digitization of medical teaching. The perceived quality of the digital infrastructure in Germany, Austria and Switzerland is good, but medical students complained about the insufficient capacity of servers at the universities; thus, digital videos can often only be viewed with a time delay and pause due to server overload. The deans also criticized the technical equipment at their universities, which lack server capacity and the technical knowledge to make full use of digital media. A study by Machado et al. showed that virtual learning platforms can become overloaded by the sheer number of medical students accessing the materials; overloading a platform causes the platform to stop functioning and impedes medical student learning [23]. A considerable number of published studies have indicated that technical difficulties exist in the implementation of digital teaching [23–29]. The lack of examinations is another weakness of virtual teaching [30, 31]. The evaluation of medical students’ opinions and their desire for self-regulated examinations are essential.

The additional time pressure created by the pandemic makes the weaknesses of digitization in medical education increasingly visible. Genuine concepts for switching to digital teaching are completely lacking. Generalization is difficult, as the prerequisites and concepts of the individual universities are very heterogeneous. There are various obstacles to mastering digitization in medical education, so hardware, software, digital skills and knowledge, as well as the desire to master this process with the appropriate time resources, must be available. However, our results also showed that in the majority of universities, digital teaching was offered for as much as 80–90% of courses only half a year after implementation. The offering of digital teaching and practical content, such as case studies, surgical techniques and examination techniques, was also confirmed by 91% of the medical students who responded to the survey, although these students viewed these offerings to be insufficient. The lecturers also confirmed this fact, with 15 of the 17 associate deans agreeing that digital teaching is currently being accelerated.

On the one hand, the survey by Loda et al. [32] postulated that medical students expected traditional teaching approaches to be transferred online. Medical students were not expected to use more innovative teaching tools. This suggests that conventional forms of teaching continue to play an important role in medical education in Germany. Based on past experiences, a mixture of reservations, technical problems, and legal requirements hindered teaching in a more innovative and creative digital way. There were also technical difficulties with the perceived poor quality of the lectures and the very one-sided implementation of face-to-face teaching with sound. This hypothesis is supported by the work of Longhurst et al. [30] and Kaup et al. [31] who showed reduced medical student engagement associated with virtual instruction.

On the other hand, many medical students see digitalization as a future prospect and appreciate the flexibility and convenience that digital teaching offers. The interface between medical students and lecturers is changing from the university campus to the digital campus. It goes without saying that the short-term restructuring of the educational infrastructure in the event of a pandemic with unprecedented societal changes cannot immediately meet all requirements [33]. This survey of medical students and lecturers showed that digital teaching is viewed positively by the majority. Medical students’ current perception of the implementation of digital teaching is only average, as is the user-friendliness of the media used and their satisfaction with the organization of digital teaching.

Understanding medical students’ perceptions of virtual instruction is an essential task to ensure effective instruction during the pandemic. 97% of medical students felt that web-based teaching was a good alternative to face-to-face teaching during the pandemic, according to Sud et al. [27]. In another study by Kaur et al. [34], this finding was confirmed among 983 medical students; these students were surveyed about their satisfaction with virtual teaching during the COVID-19 crisis. The results of the study showed that medical students perceived no qualitative difference between virtual teaching and face-to-face teaching in terms of improving communication, enhancing knowledge and skills, professional growth, and task completion. In addition, medical students were satisfied with the availability of electronic resources offered by virtual learning platforms [31]. The current literature confirms that medical students have a strong passion and determination to learn during the pandemic. Articles by Sandhu et al. [32] and Marques du Silva et al. [33] showed increased medical student participation in webinars and positive medical student feedback on web-based extracurricular lectures. Guadix et al. [34] conducted a survey to understand what medical students expect from virtual teaching in neurosurgery. Of the 127 medical students who responded, 67% wanted virtual mentoring programs and virtual surgical skills workshops in addition to the medical school curriculum [34]. Virtual teaching has become an integral part of medical education and may last even after the pandemic. Learning new skills is a challenge for which faculty members have to make time available, and technical equipment must also continue to improve. The solution to the overly theoretical approaches to digital teaching today could be provided by new digital teaching tools, such as virtual reality, digital classrooms and digital learning games. The current survey showed that medical students would like to see more innovative digital media used to reduce the main weakness of digital teaching, i.e., the lack of contact with patients, lecturers and committees, as described in other studies [31, 33, 35].

Interactive learning games, virtual reality and digital classrooms would be new innovative ways to overcome these hurdles. Through medical student feedback following a teaching format including digital rounds, it was found that 92.9% of medical students recommended this form of teaching and agreed that it stimulated learning [36]. Similarly, Murdock et al. [37] found that through the use of virtual morning rounds, medical students were provided with effective and engaging teaching. Chandra et al. [38] reported that medical students performed virtual callbacks for patients who visited the emergency department. Despite the highlighted successes of these highly interactive forms of virtual teaching, there is little literature on these programs, suggesting that they are underdeveloped and not utilized by most medical education institutions [39].

### Limitations of the study

The study provides an overview of the quality and quantity of medical education perceived by medical students and associate deans. Nonetheless, there are some limitations to the study.

The diversity of the universities reported by the students and associate deans make a general statement difficult. Much more women took part in the study, but the proportion of women in medical training was also much higher. Selection bias among students who were interested in the topic is possible.

## Conclusions

The rapid conversion of medical teaching to a digital format has been made readily and sufficiently available. Digital teaching still lacks practical relevance and the use of new digital media including learning games, virtual reality, and online questions. To meet this challenge, there is a need to improve faculty skills in teaching and demonstrations to illustrate practical clinical techniques required for a sound and indepth medical education.

## Data Availability

All data generated or analysed during this study are included in this published article.

## References

[1] Whelan A, Prescott J, Young G, Catanese V. Guidance on medical students’ clinical participation: effective immediately. Assoc Am Med Coll. 2020;17:1-6.

[2] Liang ZC, Ooi SBS, Wang W (2020). Pandemics and Their Impact on Medical Training: Lessons From Singapore. Acad med: j Assoc Amer Med Coll.

[3] Bliuc A-M, Goodyear P, Ellis RA (2007). Research focus and methodological choices in studies into students’ experiences of blended learning in higher education. Internet Higher Educ.

[4] McGarry BJ, Theobald K, Lewis PA, Coyer F (2015). Flexible learning design in curriculum delivery promotes student engagement and develops metacognitive learners: An integrated review. Nur educ today.

[5] Evans DJR, Bay BH, Wilson TD, Smith CF, Lachman N, Pawlina W (2020). Going Virtual to Support Anatomy Education: A STOPGAP in the Midst of the Covid-19 Pandemic. Anat sci educ.

[6] Ross DA (2020). Creating a “Quarantine Curriculum” to Enhance Teaching and Learning During the COVID-19 Pandemic. Acad med: j Assoc Amer Med Coll.

[7] Childs S, Blenkinsopp E, Hall A, Walton G (2005). Effective e-learning for health professionals and students–barriers and their solutions. A systematic review of the literature–findings from the HeXL project. Health info lib j.

[8] Muflih S, Abuhammad S, Karasneh R, Al-Azzam S, Alzoubi KH, Muflih M. Online Education for Undergraduate Health Professional Education during the COVID-19 Pandemic: Attitudes, Barriers, and Ethical Issues. Res Sq. 2020:rs.3.rs-42336. Preprint.

[9] Singh HK, Joshi A, Malepati RN, Najeeb S, Balakrishna P, Pannerselvam NK (2021). A survey of E-learning methods in nursing and medical education during COVID-19 pandemic in India. Nur educ today.

[10] Geha R, Dhaliwal G (2020). Pilot virtual clerkship curriculum during the COVID-19 pandemic: Podcasts, peers and problem-solving. Med educ.

[11] Mooney CJ, Peyre SE, Clark NS, Nofziger AC (2020). Rapid transition to online assessment: Practical steps and unanticipated advantages. Med educ.

[12] Kivlehan E, Chaviano K, Fetsko L, Javaid S, Chandan P, Rojas AM (2020). COVID-19 pandemic: Early effects on pediatric rehabilitation medicine training. J pedia rehab med.

[13] Hasson F, Keeney S, McKenna H (2000). Research guidelines for the Delphi survey technique. J adv nur.

[14] Turoff M (1970). The design of a policy Delphi. Techno Forecast Soc Change.

[15] Ebert JF, Huibers L, Christensen B, Christensen MB (2018). Paper- or Web-Based Questionnaire Invitations as a Method for Data Collection: Cross-Sectional Comparative Study of Differences in Response Rate, Completeness of Data, and Financial Cost. J med Internet res.

[16] Uhlig CE, Seitz B, Eter N, Promesberger J, Busse H (2014). Efficiencies of Internet-based digital and paper-based scientific surveys and the estimated costs and time for different-sized cohorts. PloS one.

[17] Khalil R, Mansour AE, Fadda WA, Almisnid K, Aldamegh M, Al-Nafeesah A (2020). The sudden transition to synchronized online learning during the COVID-19 pandemic in Saudi Arabia: a qualitative study exploring medical students’ perspectives. BMC Med Educ.

[18] Wernhart A, Gahbauer S, Haluza D (2019). eHealth and telemedicine: Practices and beliefs among healthcare professionals and medical students at a medical university. PloS one.

[19] Machleid F, Kaczmarczyk R, Johann D, Balčiūnas J, Atienza-Carbonell B, von Maltzahn F (2020). Perceptions of Digital Health Education Among European Medical Students: Mixed Methods Survey. J med Internet res.

[20] Burns KE, Duffett M, Kho ME, Meade MO, Adhikari NK, Sinuff T (2008). A guide for the design and conduct of self-administered surveys of clinicians.

[21] Iglesias C, Torgerson D (2000). Does length of questionnaire matter? A randomised trial of response rates to a mailed questionnaire. J health ser res pol.

[22] Jepson C, Asch DA, Hershey JC, Ubel PA (2005). In a mailed physician survey, questionnaire length had a threshold effect on response rate. J clin epidemiol.

[23] Machado RA, Bonan PRF, Perez D, Martelli DRB, Martelli-Júnior H (2020). I am having trouble keeping up with virtual teaching activities: Reflections in the COVID-19 era. Clin (Sao Paulo).

[24] Sahi PK, Mishra D, Singh T (2020). Medical Education Amid the COVID-19 Pandemic. Indian pedia.

[25] Atreya A, Acharya J (2020). Distant virtual medical education during COVID-19: Half a loaf of bread. clin teacher.

[26] Silva MJ, Keaveny TM, Hayes WC (1998). Computed tomography-based finite element analysis predicts failure loads and fracture patterns for vertebral sections. J ortho res: official pub Ortho Res Soc.

[27] Sud R, Sharma P, Budhwar V, Khanduja S (2020). Undergraduate ophthalmology teaching in COVID-19 times: Students’ perspective and feedback. Indian j ophthalmol.

[28] Hodgson JC, Hagan P (2020). Medical education adaptations during a pandemic: Transitioning to virtual student support. Med educ.

[29] Sahu P (2020). Closure of Universities Due to Coronavirus Disease 2019 (COVID-19): Impact on Education and Mental Health of Students and Academic Staff. Cureus.

[30] Longhurst GJ, Stone DM, Dulohery K, Scully D, Campbell T, Smith CF, Strength (2020). Weakness, Opportunity, Threat (SWOT) Analysis of the Adaptations to Anatomical Education in the United Kingdom and Republic of Ireland in Response to the Covid-19 Pandemic. Anat sci educ.

[31] Kaup S, Jain R, Shivalli S, Pandey S, Kaup S (2020). Sustaining academics during COVID-19 pandemic: The role of online teaching-learning. Indian j ophthalmol.

[32] Loda T, Löffler T, Erschens R, Zipfel S, Herrmann-Werner A (2020). Medical education in times of COVID-19: German students’ expectations - A cross-sectional study. PloS one.

[33] Mian A, Khan S (2020). Medical education during pandemics: a UK perspective. BMC med.

[34] Kaur N, Dwivedi D, Arora J, Gandhi A (2020). Study of the effectiveness of e-learning to conventional teaching in medical undergraduates amid COVID-19 pandemic. Nat J Physiol, Pharma Pharmacol.

[35] Dedeilia A, Sotiropoulos MG, Hanrahan JG, Janga D, Dedeilias P, Sideris M (2020). Medical and Surgical Education Challenges and Innovations in the COVID-19 Era: A Systematic Review. In vivo (Athens, Greece)..

[36] Hofmann H, Harding C, Youm J, Wiechmann W (2020). Virtual bedside teaching rounds with patients with COVID-19. Med educ.

[37] Murdock HM, Penner JC, Le S, Nematollahi S (2020). Virtual Morning Report during COVID-19: A novel model for case-based teaching conferences. Med educ.

[38] Chandra S, Laoteppitaks C, Mingioni N, Papanagnou D (2020). Zooming-out COVID-19: Virtual clinical experiences in an emergency medicine clerkship. Med educ.

[39] Wilcha R-J (2020). Effectiveness of Virtual Medical Teaching During the COVID-19 Crisis: Systematic Review. JMIR Med Educ.

